# The Amino Acid Transporter Mct10/Tat1 Is Important to Maintain the TSH Receptor at Its Canonical Basolateral Localization and Assures Regular Turnover of Thyroid Follicle Cells in Male Mice

**DOI:** 10.3390/ijms22115776

**Published:** 2021-05-28

**Authors:** Vaishnavi Venugopalan, Alaa Al-Hashimi, Jonas Weber, Maren Rehders, Maria Qatato, Eva K. Wirth, Ulrich Schweizer, Heike Heuer, François Verrey, Klaudia Brix

**Affiliations:** 1 Department of Life Sciences and Chemistry, Focus Area HEALTH, Jacobs University Bremen, Campus Ring 1, D-28759 Bremen, Germany; v.venugopalan@jacobs-university.de (V.V.); a.alhashimi@jacobs-university.de (A.A.-H.); jonas.weber@perkinelmer.com (J.W.); m.rehders@jacobs-university.de (M.R.); m.qatato@dkfz-heidelberg.de (M.Q.); 2 Berlin Institute of Health, Department of Endocrinology and Metabolism, Charité-Universitätsmedizin Berlin, Corporate Member of Freie Universität Berlin, Humboldt-Universität zu Berlin and DZHK (German Centre for Cardiovascular Research), Partner Site Berlin, Hessische Str. 3-4, D-10115 Berlin, Germany; eva.wirth@charite.de; 3 Institut für Biochemie und Molekularbiologie, Universitätsklinikum Bonn, Nußallee 11, D-53115 Bonn, Germany; uschweiz@uni-bonn.de; 4 Department of Endocrinology, Diabetes and Metabolism, University of Duisburg-Essen, Universitätsklinikum Essen, Hufelandstr. 55, D-45147 Essen, Germany; heike.heuer@uk-essen.de; 5 Physiologisches Institut, Universität Zürich, Winterthurerstr. 190, CH-8057 Zürich, Switzerland; francois.verrey@uzh.ch

**Keywords:** monocarboxylate transporter 10/Scl16a10, thyroid gland architecture, thyrocyte survival, TSH receptor signaling

## Abstract

Cathepsin K-mediated thyroglobulin proteolysis contributes to thyroid hormone (TH) liberation, while TH transporters like Mct8 and Mct10 ensure TH release from thyroid follicles into the blood circulation. Thus, thyroid stimulating hormone (TSH) released upon TH demand binds to TSH receptors of thyrocytes, where it triggers Gα_q_-mediated short-term effects like cathepsin-mediated thyroglobulin utilization, and Gα_s_-mediated long-term signaling responses like thyroglobulin biosynthesis and thyrocyte proliferation. As reported recently, mice lacking Mct8 and Mct10 on a cathepsin K-deficient background exhibit excessive thyroglobulin proteolysis hinting towards altered TSH receptor signaling. Indeed, a combination of canonical basolateral and non-canonical vesicular TSH receptor localization was observed in *Ctsk*^−/−^/*Mct8*^−/y^/*Mct10*^−/−^ mice, which implies prolonged Gα_s_-mediated signaling since endo-lysosomal down-regulation of the TSH receptor was not detected. Inspection of single knockout genotypes revealed that the TSH receptor localizes basolaterally in *Ctsk*^−/−^ and *Mct8*^−/y^ mice, whereas its localization is restricted to vesicles in *Mct10*^−/−^ thyrocytes. The additional lack of cathepsin K reverses this effect, because *Ctsk*^−/−^/*Mct10*^−/−^ mice display TSH receptors basolaterally, thereby indicating that cathepsin K and Mct10 contribute to TSH receptor homeostasis by maintaining its canonical localization in thyrocytes. Moreover, *Mct10*^−/−^ mice displayed reduced numbers of dead thyrocytes, while their thyroid gland morphology was comparable to wild-type controls. In contrast, *Mct8*^−/y^, *Mct8*^−/y^/*Mct10*^−/−^, and *Ctsk*^−/−^/*Mct8*^−/y^/*Mct10*^−/−^ mice showed enlarged thyroid follicles and increased cell death, indicating that Mct8 deficiency results in altered thyroid morphology. We conclude that vesicular TSH receptor localization does not result in different thyroid tissue architecture; however, Mct10 deficiency possibly modulates TSH receptor signaling for regulating thyrocyte survival.

## 1. Introduction

The thyroid gland functions to supply the thyroid hormones (TH) triiodothyronine (T3) and thyroxine (T4) to almost all tissues of the body, a process that is enabled by proteolytic cleavage of the prohormone thyroglobulin (Tg) by cathepsins B, D, K, L, and S [1]. Subsequently, the transport of TH from thyroid follicles into the blood circulation is mediated by TH transmembrane transporters such as monocarboxylate transporters Mct8 (Slc16a2) and Mct10 (Slc16a10) [2]. TH provide master endocrine control in mammals from the embryonic stage through adulthood and ageing, hence necessitating regulation at the level of the thyroid gland regarding TH generation, liberation, and release [1,3]. The classical pathway of thyroid gland regulation via the hypothalamus–pituitary–thyroid (HPT) axis encompasses triggering thyroid stimulating hormone (TSH) receptor signaling to thyrocytes by binding of its ligand TSH, a glycoprotein hormone of the pituitary, that itself is regulated by negative feedback upon TH demand [4,5]. The TSH receptor belongs to the G-protein coupled receptors (GPCR), whereby TSH-induced signaling regulates Tg turnover through Gα_s_-mediated long-term and Gα_q_-mediated short-term responses that respectively balance the repetitive cycles of Tg biosynthesis and Tg proteolysis for TH liberation [3].

Recently, we have reported that mice lacking Mct8 and Mct10 on a cathepsin K-deficient background exhibit autophagy-induced excessive cathepsin-mediated Tg proteolysis [6]. The resulting enhanced intrathyroidal TH accumulation, due to the lack of exporting TH transporters, leads to self-toxicity in thyrocytes of *Ctsk*^−/−^/*Mct8*^−/y^/*Mct10*^−/−^ mice. Such a phenotype is also observed in mice lacking Mct8 and cathepsin K, but not in a combined Mct10- and cathepsin K-deficient genotype [6]. This indicates that while Tg utilization is possibly induced in *Ctsk*^−/−^/*Mct8*^−/y^ and *Ctsk*^−/−^/*Mct8*^−/y^/*Mct10*^−/−^, it appears unaffected in the *Ctsk*^−/−^/*Mct10*^−/−^ mice, where indeed cathepsin-mediated Tg degradation remains unchanged from wild-type (WT) controls. However, *Ctsk*^−/−^/*Mct10*^−/−^ mice show reduced total Tg amounts and the extent of Tg glycosylation is diminished when compared to WT controls. Consequently, *Ctsk*^−/−^/*Mct10*^−/−^ thyroid follicles display enhanced amounts of soluble Tg, hinting at deregulated Tg biosynthesis, where reduced glycosylation results in less stable Tg deposits in the thyroid follicle lumen [6]. Since Tg biosynthesis is regulated via Gα_s_, while Tg proteolysis is mediated by Gα_q_, we suggest that intrathyroidal TSH receptor signaling might be altered in TH transporter deficiencies, specifically on a cathepsin K-deficient background, resulting in altered thyroid functional phenotypes. 

An important determinant of the outcomes of TSH receptor signaling for maintenance of normal thyroid gland function is the size of the thyroid gland, which is attributed to several parameters, including the number of thyroid follicles, area and number of thyrocytes, epithelial height, area occupied by the follicle lumen, and the area occupied by or numbers of other cell types residing within the thyroid gland [6,7,8]. Therefore, understanding thyroid gland structure-to-function relationships is critical for proper evaluation and treatment of thyroid dysfunctional states [9]. For instance, mutations in X-chromosomal *Mct8* result in severe thyroid pathologies with nuclear characteristics of papillary thyroid carcinoma (PTC), indicating dedifferentiation of thyrocytes in male Mct8-deficient animals (*Mct8*^−/y^) that leads to massive thyrocyte hyperproliferation [10]. The occurrence of such hyperplasia phenotypes argues for prolonged Gα_s_-mediated TSH receptor signaling in *Mct8*^−/y^ mice [10,11]. However, a role of Gα_q_-mediated TSH receptor signaling in hyperplasia-resulting thyrocyte proliferation cannot be ruled out [12]. On the contrary, genotypes featuring upregulation of Mct8 such as that observed in *Ctsk*^−/−^ mice [8] result neither in altered thyroid functional nor morphological phenotypes [13,14]. Although it is known that serum TSH concentrations remain unaffected in *Ctsk*^−/−^ mice [14] and are significantly [10] or moderately elevated in Mct8 deficiency [11,15,16] in young or adult mice, respectively, no information is available on the outcomes of TSH receptor signaling in genotypes where Mct8 is lacking (i.e., *Mct8*^−/y^) or upregulated (i.e., *Ctsk*^−/−^). Moreover, in-depth knowledge on whether differences in TSH receptor localization can impact signaling, and, consequently, thyroid gland morphology and physiology, is still at the beginning of our understanding [17,18].

The TSH receptor is canonically located at the basolateral plasma membrane of thyrocytes, however, recent studies have shown that ligand binding leads to the internalization of TSH-TSH receptor complexes, whereupon Gα_s_ signaling from late endo-lysosomal compartments can persist [19]. This suggests that aside from serum TSH concentrations, the localization of TSH receptors can be an additional factor in determining the downstream effects of TSH-triggered TSH receptor signaling [17,18]. In addition, the TSH receptor has the ability to oligomerize with itself [20,21] and with the TH transporter Mct8, thereby potentially further modulating the outcomes of signaling [3,22]. By way of example, in vitro studies with HEK cells demonstrated that co-expressing Mct8 and TSH receptor dampens TSH-stimulated Gα_q_ signaling, while cAMP accumulation upon Gα_s_ signaling is only slightly affected [22]. Whether other TH transporters such as the amino acid transporter Mct10/Tat1 can similarly interact with the TSH receptor and whether such an interaction would result in biased signaling in vivo is not known but is approached in this study by using TH transporter-deficient mice.

Consequently, in the present study, the localization of the TSH receptor was assessed in thyroid tissue of *Ctsk*^−/−^, *Mct10*^−/−^, *Mct8*^−/y^, *Mct8*^−/y^/*Mct10*^−/−^, *Ctsk*^−/−^/*Mct10*^−/−^, *Ctsk*^−/−^/*Mct8*^−/y^, and *Ctsk*^−/−^/*Mct8*^−/y^/*Mct10*^−/−^ male mice in comparison with WT controls. Then, epithelial extensions and distribution of intra- vs. extracellular cathepsins were determined as a proxy of Gα_q_ signaling, while the determination of follicle numbers, follicle and lumen areas, thyrocyte areas and numbers, and follicular cathepsin amounts were studied as a proxy of Gα_s_-mediated signaling. Because some of these parameters, for instance cathepsin-mediated Tg utilization, were previously analyzed in *Ctsk*^−/−^, *Mct10*^−/−^, *Mct8*^−/y^, and *Mct8*^−/y^/*Mct10*^−/−^ mice [6,8,13,14], here we focused on inspecting the *Ctsk*^−/−^/*Mct10*^−/−^, *Ctsk*^−/−^/*Mct8*^−/y^, and *Ctsk*^−/−^/*Mct8*^−/y^/*Mct10*^−/−^ genotypes more comprehensively than before [6]. Our data indicate a possible role of Mct10 in maintaining the TSH receptor at its canonical basolateral localization. TSH receptor delocalization in Mct10 deficiency is overcome by a combined cathepsin K deficiency in *Ctsk*^−/−^/*Mct10*^−/−^ and *Ctsk*^−/−^/*Mct8*^−/y^/*Mct10*^−/−^ mice but not observed in cathepsin K deficiency alone. Furthermore, we report that vesicular TSH receptor localization does not lead to gross alterations in thyroid morphology, but balanced TSH receptor signaling is essential to maintain regular thyrocyte numbers, because Mct10 deficiency results in reduced thyrocyte death rates.

## 2. Results

### 2.1. Serum TSH Concentrations in Mice Lacking Cathepsin K and TH Transporters

**Figure 1:**
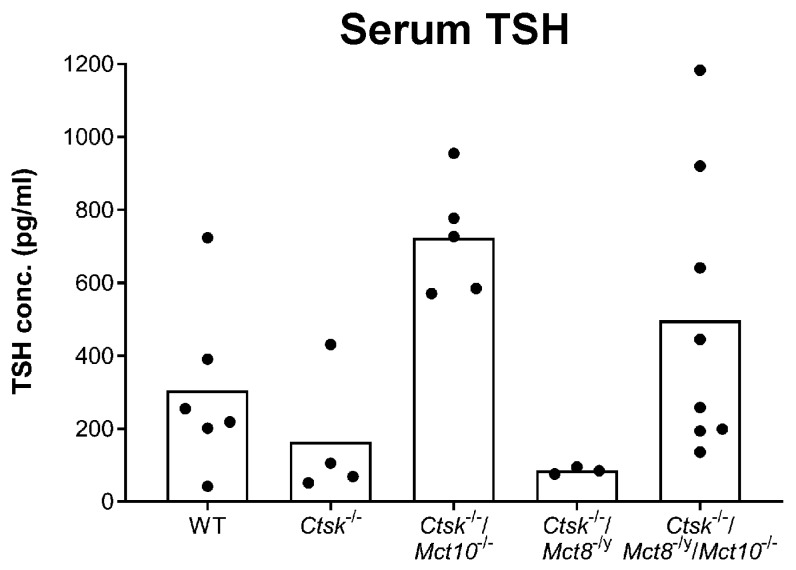
TSH concentration in the blood sera of mice lacking cathepsin K and/or TH transporters. Serum samples prepared from mice of the indicated genotypes were subjected to ELISA for determining TSH concentrations. Scatter graphs displaying serum TSH concentrations as individual readings and as bars indicating the means showed no statistically significant genotypic differences when tested by Dunnett’s multiple comparison analysis. Animals analyzed: *n* = 6, 4, 5, 3, 8 for WT, *Ctsk*^−/−^, *Ctsk*^−/−^/*Mct10*^−/−^, *Ctsk*^−/−^/*Mct8*^−/y^, and *Ctsk*^−/−^/*Mct8*^−/y^/*Mct10*^−/−^, respectively.

In order to address whether enhanced Tg proteolysis in *Ctsk*^−/−^/*Mct8*^−/y^ and *Ctsk*^−/−^/*Mct8*^−/y^/*Mct10*^−/−^ thyroid glands and decreased amounts of total thyroidal Tg in *Ctsk*^−/−^/*Mct10*^−/−^ mice is due to potential differences in HPT axis regulation in *Mct8* vs. *Mct10* deficiency, blood serum collected from male WT, *Ctsk*^−/−^, *Ctsk*^−/−^/*Mct10*^−/−^, *Ctsk*^−/−^/*Mct8*^−/y^, and *Ctsk*^−/−^/*Mct8*^−/y^/*Mct10*^−/−^ mice was analyzed using mouse TSH-specific ELISA (Figure 1). Mice deficient in cathepsin K showed TSH comparable to WT controls, which is in accordance with our previously published data [14]. A decrease in the average serum TSH concentration was observed when comparing *Ctsk*^−/−^/*Mct8*^−/y^ mice with their WT counterparts. In contrast, *Mct10* deficiency on a cathepsin K background. i.e., *Ctsk*^−/−^/*Mct10*^−/−^ and *Ctsk*^−/−^/*Mct8*^−/y^/*Mct10*^−/−^, led to an increase in serum TSH concentrations of approximately 2-fold in comparison to young WT controls (Figure 1). However, these results did not reach statistical significance through Dunnett’s multiple comparison analysis. 

These results are in support of our recently proposed conclusion that the triple-deficient murine model displayed altered thyroid phenotypes through serum TSH-independent pathways [6]. 

### 2.2. Localization of TSH Receptors in Thyroid Glands of Combined Cathepsin K and/or TH Transporter Deficiency

**Figure 2:**
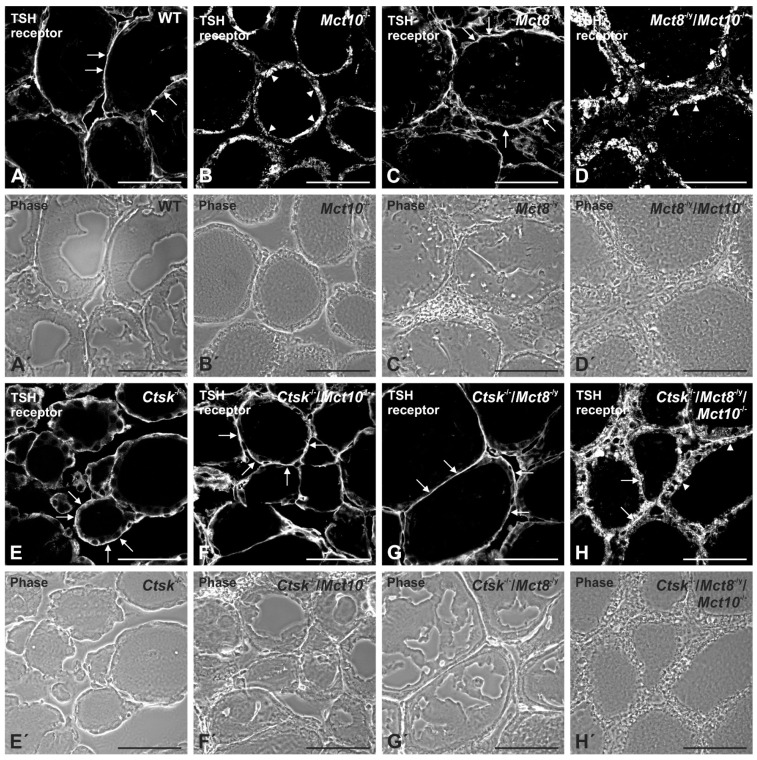
TSH receptor localization in cathepsin K and/or TH transporter deficiency. Thyroid cryosections from mice of the indicated genotypes and WT controls were stained with antibodies specific for TSH receptor (**A**–**H**, white). Corresponding phase contrast micrographs are displayed as indicated (**A’**–**H’**). Micrographs show that the TSH receptor localizes canonically to the basolateral plasma membrane (arrows) in WT controls, *Ctsk*^−/−^, *Ctsk*^−/−^/*Mct10*^−/−^, and *Ctsk*^−/−^/*Mct8*^−/y^ thyrocytes (**A**,**E**–**G**, respectively). Inspection of single TH transporter knockout genotypes (**B**,**C**) revealed that TSH receptor localizes to vesicles when Mct10 alone is lacking (**B**, arrowheads), and this effect was further confirmed in *Mct8*^−/y^/*Mct10*^−/−^ mice that showed vesicular TSH receptor localization (**D**, arrowheads). On the contrary, a combination of basolateral (arrows) and vesicular TSH receptor localization (arrowheads) was observed in *Ctsk*^−/−^/*Mct8*^−/y^/*Mct10*^−/−^ mice (**H**). Animals analyzed: *n* = 3 per genotype with 8–10 micrographs quantified per animal, respectively. Scale bars represent 50 µm.

Thyroid gland regulation by the HPT axis depends on both serum TSH concentration as well as TSH receptor localization as shown previously for rodents [17]. Therefore, tissue sections from the thyroid glands of WT, *Ctsk*^−/−^, *Mct10*^−/−^, *Mct8*^−/y^, *Mct8*^−/y^/*Mct10*^−/−^, *Ctsk*^−/−^/*Mct10*^−/−^, *Ctsk*^−/−^/*Mct8*^−/y^, and *Ctsk*^−/−^/*Mct8*^−/y^/*Mct10*^−/−^ mice were morphologically analyzed by immunofluorescence using TSH receptor-specific antibodies (Figure 2). Micrographs showed that while TSH receptors localized basolaterally in WT controls, *Ctsk*^−/−^ and *Mct8*^−/y^ thyrocytes (Figure 2A,C,E, respectively, arrows), this canonical distribution of TSH receptors was absent in the thyroid glands of *Mct10*^−/−^ mice (Figure 2B). Instead, *Mct10*^−/−^ thyrocytes showed TSH receptors exclusively in vesicles (Figure 2B, arrowheads), suggesting that Mct10 is important in keeping TSH receptors in their classical basolateral location. To confirm this conclusion, the TSH receptor localization was evaluated in *Mct8*^−/y^/*Mct10*^−/−^ mice, which revealed predominantly vesicular localization and the absence of basolateral localization (Figure 2D, arrowheads). It was further revealed that in genotypes where either Mct10 or Mct8 was lacking on a cathepsin-K deficient background, the classical TSH receptor localization, i.e., at the basolateral plasma membrane of thyrocytes, was maintained (Figure 2F,G, respectively, arrows), indicating that the additional deficiency of cathepsin K outcompetes vesicular TSH receptor localization observed in Mct10 deficiency. However, triple-deficiency led to altered localization patterns wherein TSH receptors were found additionally in vesicular compartments (Figure 2H, arrowheads) aside from the basolateral plasma membrane (Figure 2H, arrows).

Taken together, we conclude that lack of Mct10 affects TSH receptor localization, which may be stipulated as altered TSH receptor signaling, possibly leading to an altered intrathyroidal response to TSH stimuli and persistent Gα_s_ signaling in Mct10 deficiency.

### 2.3. Assessing Endocytic Down-Regulation of TSH Receptors in Combined Cathepsin K and TH Transporter Deficiencies

**Figure 3:**
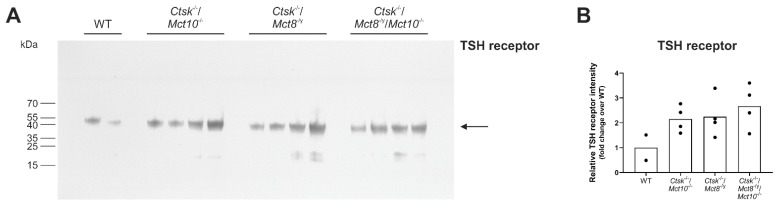
Integrity of TSH receptors in cathepsin K and TH transporter deficiencies. Proteins isolated from whole thyroid tissue lysates of the indicated genotypes and WT controls were separated on 8–18% horizontal SDS-gels, transferred onto nitrocellulose membrane, and immunoblotted using TSH receptor-specific antibodies. A representative immunoblot is shown (**A**). The molecular mass markers are given in the left margin. TSH receptor band intensities were determined by densitometry and normalized to total Ponceau-stained protein per lane (see Supplementary Figure S1). Bar charts (**B**) represent densitometry analyses of the TSH receptor band intensities in the investigated genotypes as fold changes over WT controls as means, while symbols indicate data points of individual animals. Significant genotypic differences in band intensities of TSH receptors were not observed. In addition, no obvious TSH receptor degradation fragments were observed indicating that TSH receptors were intact despite endo-lysosomal localization. Animals analyzed: *n* = 2–4 per genotype.

In order to study whether vesicular TSH receptors observed in triple-deficient mice are en route for endo-lysosomal degradation or are stable upon internalization, whole thyroid tissue lysates from *Ctsk*^−/−^/*Mct10*^−/−^, *Ctsk*^−/−^/*Mct8*^−/y^, and *Ctsk*^−/−^/*Mct8*^−/y^ /*Mct10*^−/−^ mice and WT controls were analyzed by immunoblotting using antibodies specific for TSH receptor (Figure 3A). Quantification of the immuno-recognized bands by densitometry revealed no differences in TSH receptor representative band intensities in any genotype when compared to WT controls (Figure 3B). Furthermore, low molecular mass bands, reminiscent of protein fragments, were not prominent amongst the investigated genotypes, thereby suggesting that TSH receptor downregulation by degradation was not prevalent. Together, vesicular TSH receptors in *Ctsk*^−/−^/*Mct8*^−/y^/*Mct10*^−/−^ thyrocytes are most likely intact and intracellular TSH-stimulated receptor signaling can thus, in principle, persist for prolonged time periods.

### 2.4. Sub-Follicular Cathepsin Distribution Is Unaltered in Combined Cathepsin K- and TH Transporter-Deficient Mice

In response to TSH-stimulated short-term TSH receptor signaling, cathepsins travel from endocytic compartments to the apical plasma membrane and are subsequently secreted into the thyroid follicle lumen [23,24]. Thus, a read-out for Gα_q_-mediated TSH receptor signaling is obtained by analyzing extra- vs. intracellular cathepsin distribution in thyroid follicles. Therefore, we studied the sub-follicular distribution and the sub-cellular localization of cathepsins in the mouse models lacking cathepsin K and TH transporters to examine whether Gα_q_-mediated signaling outcomes may be altered in these genotypes.

**Figure 4:**
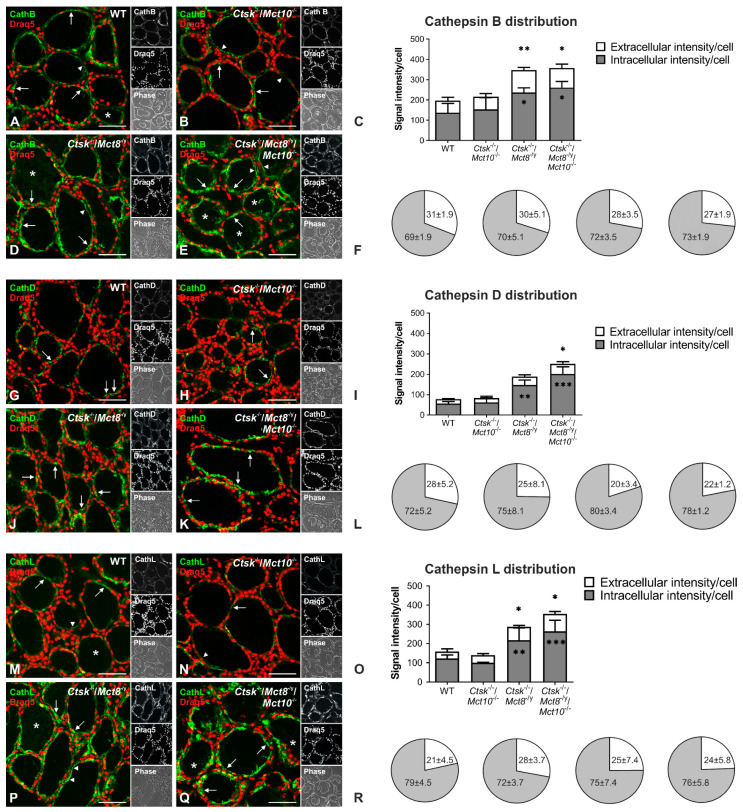
Localization and distribution of cathepsins B, D, and L in thyroid glands lacking cathepsin K and TH transporters. Thyroid cryosections from WT, *Ctsk*^−/−^/*Mct10*^−/−^, *Ctsk*^−/−^/*Mct8*^−/y^, and *Ctsk*^−/−^/*Mct8*^−/y^/*Mct10*^−/−^ mice were stained with antibodies (green) against cathepsin B (**A**,**B**,**D**,**E**), cathepsin D (**G**,**H**,**J**,**K**), or cathepsin L (**M**,**N**,**P**,**Q**). Merged, single-channel fluorescence and corresponding phase contrast micrographs are displayed as indicated. Cathepsins B, D, and L localized intracellularly within vesicular structures (arrows) and in the thyroid follicle lumen (asterisks) or re-associated with the apical plasma membrane of thyrocytes (arrowheads) in all investigated genotypes. The intensity of cathepsin B, D, and L staining (**C**,**I**,**O**, respectively) was measured in the extracellular lumen and in the apical pericellular region (white bars) or within thyrocytes (grey bars) using a Cell Profiler-based pipeline and normalized to the numbers of cells. Bars graphs show means + SD. In *Ctsk*^−/−^/*Mct8*^−/y^ and *Ctsk*^−/−^/*Mct8*^−/y^/*Mct10*^−/−^ thyroid glands, the total intensity of cathepsin B, D, and L signals were enhanced in comparison to WT controls. However, no significant genotypic differences were observed in the ratios of intracellular (grey) or extracellular (white) cathepsin signals over respective total cathepsin signals, given in percentages (**F**,**L**,**R**, represented as pie charts). Animals analyzed: *n* = 3 per genotype with 7–12 micrographs quantified per animal, respectively. Nuclei were counter-stained with Draq5^TM^ (red). Scale bars represent 50 µm. Numbers in pie charts are depicted as means ± SD. Data are given as fold-changes over WT controls (in bar graphs) and as percentages (in pie charts). Levels of significance are indicated as * for *p* < 0.05, ** for *p* < 0.01, and *** for *p* < 0.001.

Thyroid tissue sections were stained with antibodies against cathepsins B, D, or L, and analyzed by confocal laser scanning microscopy. Cathepsins B, D, and L mainly localized within vesicular compartments of thyrocytes in WT, *Ctsk*^−/−^/*Mct10*^−/−^, *Ctsk*^−/−^/*Mct8*^−/y^, and *Ctsk*^−/−^/*Mct8*^−/y^/*Mct10*^−/−^ mice (Figure 4, arrows), while in some follicles, cathepsins were additionally detected in the follicle lumen (asterisks). Furthermore, cathepsin signals were also found pericellularly due to the re-association of intraluminal cathepsins with the apical plasma membrane domain of thyrocytes (Figure 4, arrowheads). Overall, immunofluorescence intensities of thyroidal cathepsins B, D, and L in *Ctsk*^−/−^/*Mct10*^−/−^ were equal to WT when determined on a per-cell basis (Figure 4C,I,O); the cathepsin protein amounts were enhanced in *Ctsk*^−/−^/*Mct8*^−/y^ and *Ctsk*^−/−^/*Mct8*^−/y^/*Mct10*^−/−^ (Figure 4C,I,O), which is in line with the aforementioned autophagy induction upon *Mct8* loss [6]. The proportions of intracellular vs. extracellular (sum of luminal and pericellular) cathepsins in WT mice were 69% ± 1.9% vs. 31% ± 1.9% for cathepsin B, 72% ± 5.2% vs. 28% ± 5.2% for cathepsin D, and 79% ± 4.5% vs. 21% ± 4.5% for cathepsin L (Figure 4F,L,R, leftmost pie-chart). Thus, although per-cell signal intensity measurements of luminal and pericellular cathepsins B, D, and L were increased in *Ctsk*^−/−^/*Mct8*^−/y^ and *Ctsk*^−/−^/*Mct8*^−/y^ /*Mct10*^−/−^ mice in comparison to WT controls (Figure 4C,I,O, white bars), the proportion of extracellular cathepsin signal relative to the respective total intensity remained unchanged in all genotypes (Figure 4F,L,R). Similarly, despite the enhanced endo-lysosomal per-cell signals of cathepsins B, D, and L in *Ctsk*^−/−^/*Mct8*^−/y^ and *Ctsk*^−/−^/*Mct8*^−/y^/*Mct10*^−/−^ mice when compared to WT controls (Figure 4C,I,O, grey bars), the ratios of intracellular signal intensity over total signals for the respective cathepsins were comparable to those from WT controls (Figure 4F,L,R) in all genotypes. These results point to the notion that increased secretion of thyroidal cathepsins into the extracellular follicle lumen as a result of prolonged short-term TSH receptor signaling in *Ctsk*^−/−^/*Mct8*^−/y^/*Mct10*^−/−^ mice is not prevalent.

### 2.5. Measurement of Epithelial Heights in Cathepsin K and/or TH Transporter Deficiency

**Figure 5:**
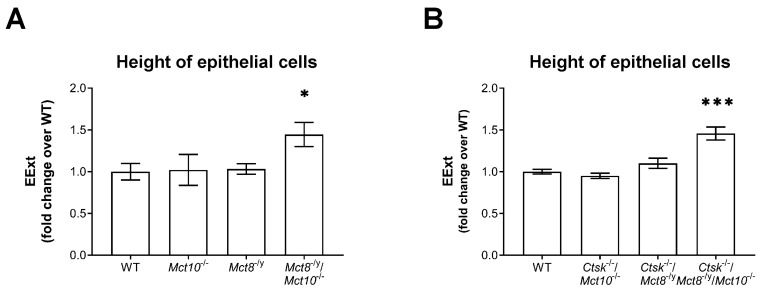
Height of the thyroid follicular epithelia in cathepsin K and/or TH transporter deficiency. Thyroid tissue mid-sections from indicated genotypes were analyzed by a Cell Profiler-based pipeline to determine the heights of thyrocytes, i.e., epithelial extensions (EExt) (**A**,**B**). Note that EExts are higher in genotypes where both Mct8 and Mct10 are lacking when compared to the WT controls. Increasingly prismatic thyrocytes suggest increased activity of the thyroid glands of *Mct8*^−/y^/*Mct10*^−/−^ and *Ctsk*^−/−^/*Mct8*^−/y^/*Mct10*^−/−^ mice. Animals analyzed: *n* = 3–9 per genotype. No. of follicles analyzed: *n* = 1569, 890, 1105, 614, 422, 552, and 630 for WT, *Mct10*^−/−^, *Mct8*^−/y^, *Mct8*^−/y^/*Mct10*^−/−^, *Ctsk*^−/−^/*Mct10*^−/−^, *Ctsk*^−/−^/*Mct8*^−/y^, and *Ctsk*^−/−^/*Mct8*^−/y^/*Mct10*^−/−^, respectively. Note that the analyses were performed with different animal cohorts, therefore the data are depicted in comparison to the specific WT controls of each cohort and given as fold changes over WT controls as means ± SD. Levels of significance are indicated as * for *p* < 0.05 and *** for *p* < 0.001.

Epithelial extensions (EExt) indicate thyroid follicle activity and hence reflect the short-term TSH receptor signaling pathway mediated by Gα_q_. Therefore, in order to appraise the genotypic differences in the epithelial heights of thyrocytes, thyroid mid-sections from WT, *Mct10*^−/−^, *Mct8*^−/y^, *Mct8*^−/y^/*Mct10*^−/−^, *Ctsk*^−/−^/*Mct10*^−/−^, *Ctsk*^−/−^/*Mct8*^−/y^, and *Ctsk*^−/−^/*Mct8*^−/y^/*Mct10*^−/−^ mice stained with anti-collagen IV or anti-Laminin 1+2 antibodies, CMO, and Draq5™ were analyzed using a Cell Profiler-based pipeline (see Section 4.5.2. below). Results showed that in comparison to WT controls, EExt was significantly increased only when both Mct8 and Mct10 are missing, i.e., in *Mct8*^−/y^/*Mct10*^−/−^ and *Ctsk*^−/−^/*Mct8*^−/y^/*Mct10*^−/−^ mice (Figure 5A,B, respectively). In contrast, thyrocytes lacking either Mct8 or Mct10 displayed average EExt comparable to WT thyrocytes, irrespective of cathepsin K functionality (Figure 5). Previously, it was found that EExt in cathepsin K deficiency are also comparable to WT thyrocytes [14]. Since it is known that prismatic thyrocytes or follicles with a more polarized epithelium show enhanced function compared to thyrocytes within a flat epithelium, our data comply with our previous findings that cathepsin-mediated Tg degradation is most pronounced in *Mct8*^−/y^/*Mct10*^−/−^ [8] and *Ctsk*^−/−^/*Mct8*^−/y^/*Mct10*^−/−^ [6] when compared to all inspected genotypes. These results indicate that an obvious correlation between TSH receptor localization and epithelial height is lacking, given that EExt remained unaffected in *Mct10*^−/−^ mice that feature vesicular TSH receptors (cf. Figure 2). 

### 2.6. Assessing Thyroid Gland Architecture by Morphometry in Combined Cathepsin K and/or TH Transporter Deficiencies

**Figure 6:**
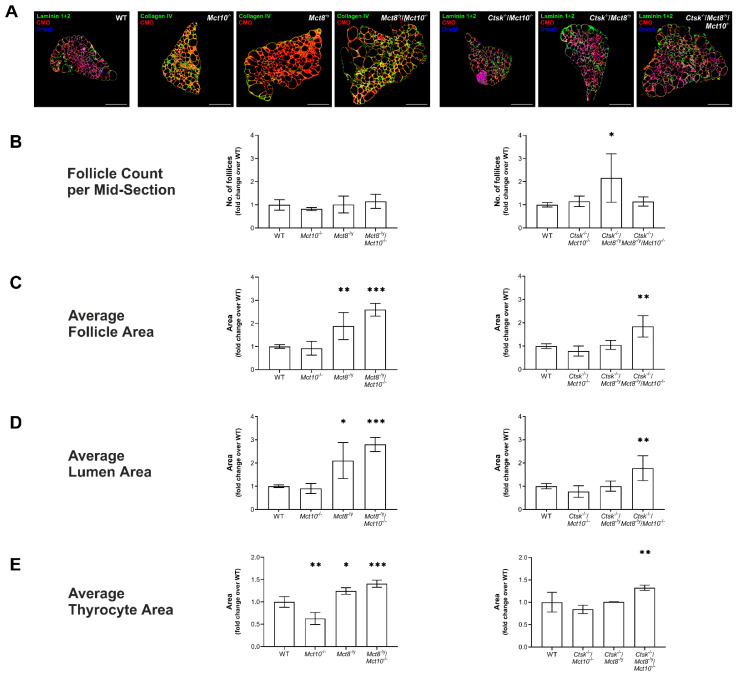
Morphometry of thyroid lobes in cathepsin K and/or TH transporter deficiency. Thyroid tissue cryosections of indicated genotypes incubated with anti-Laminin 1 + 2 and anti-Collagen IV antibodies (respectively, green), CMO (red), and Draq5^TM^ (blue) were analyzed by a Cell Profiler-based pipeline. A representative whole thyroid lobe per genotype is shown (**A**). Bar graphs depicting follicle counts per thyroid mid-section (**B**), average follicle area (**C**), average lumen area (**D**), and average thyrocyte area (**E**) are shown. A 2-fold increase in follicle numbers was observed in *Ctsk*^−/−^/*Mct8*^−/y^ in comparison to WT controls (**B**). A significant increase in average follicle area, lumen area, and thyrocyte areas was observed in *Mct8*^−/y^, *Mct8*^−/y^/*Mct10*^−/−^, and *Ctsk*^−/−^/*Mct8*^−/y^/*Mct10*^−/−^ mice (**B**–**E**, respectively) when compared to WT controls. Note the reduced average thyrocyte area in *Mct10*^−/−^ mice (**E**, left panel). Follicle area is defined as the external edge of the follicular epithelia, i.e., the Laminin 1 + 2- or Collagen IV-positive basal lamina surrounding the thyroid follicle lumen. Lumen area is measured upon inverting the CMO-stained thyroid epithelium to identify the thyroid follicle lumen. Thyrocyte area is measured by subtracting lumen area from follicle area and dividing by the number of cells per follicle. Scale bars represent 500 µm. Animals analyzed: *n* = 3–9 per genotype. No. of follicles analyzed: *n* = 1569, 890, 1105, 614, 422, 552, and 630 for WT, *Mct10^−/−^, Mct8*^−/y^, *Mct8*^−/y^/*Mct10*^−/−^, *Ctsk*^−/−^/*Mct10*^−/−^, *Ctsk*^−/−^/*Mct8*^−/y^, and *Ctsk*^−/−^/*Mct8*^−/y^/*Mct10*^−/−^, respectively. Note that the analyses were performed with different animal cohorts, therefore the data are depicted in comparison to the specific WT controls of each cohort and given as fold changes over WT controls as means ± SD. Levels of significance are indicated as * for *p* < 0.05, ** for *p* < 0.01, and *** for *p* < 0.001.

In order to understand whether altered TSH receptor localization (cf. Figure 2) affects thyroid gland architecture in the long term, we determined the number of thyroid follicles, average follicle area, average lumen area, and average thyrocyte area in the thyroid glands of WT, *Mct10*^−/−^, *Mct8*^−/y^, *Mct8*^−/y^/*Mct10*^−/−^, *Ctsk*^−/−^/*Mct10*^−/−^, *Ctsk*^−/−^/*Mct8*^−/y^, and *Ctsk*^−/−^/*Mct8*^−/y^/*Mct10*^−/−^ mice (Figure 6), while our previous studies already revealed no alterations on the determined parameters in *Ctsk*^−/−^ mice [14]. Thyroid cryosections were immunolabelled with anti-Collagen IV or anti-Laminin 1 + 2 antibodies to determine follicle boundaries, while the epithelial cells were stained with CMO, and Draq5™-counterstained nuclei were used to enumerate cells, before imaging by confocal laser scanning microscopy (Figure 6A) and using Cell Profiler pipeline P2 to quantify intended measurements. 

Morphological assessment revealed that the number of thyroid follicles per lobe in *Ctsk*^−/−^/*Mct8*^−/y^ showed a significant two-fold increase when compared to WT thyroid tissue mid-section, while no changes in follicle counts were found in the other genotypes (Figure 6B). Interestingly, in comparison to WT tissue, an increase in the average follicle area was observed only in the *Mct8*^−/y^, *Mct8*^−/y^/*Mct10*^−/−^, and *Ctsk*^−/−^/*Mct8*^−/y^/*Mct10*^−/−^ mice, while no significant differences were seen in *Mct10*^−/−^, *Ctsk*^−/−^/*Mct10*^−/−^ and *Ctsk*^−/−^/*Mct8*^−/y^ mice (Figure 6C). It is important to note that the size of the follicle lumen relates to the amounts of extracellular Tg, both cross-linked and partially processed Tg, and is indicative of the balance between Tg deposition and Tg proteolysis. Results revealed that the average follicle lumen area in mid-sections from *Mct8*^−/y^, *Mct8*^−/y^/*Mct10*^−/−^, and *Ctsk*^−/−^/*Mct8*^−/y^/*Mct10*^−/−^ thyroid glands displayed 2- to 3-fold increases in luminal area in comparison to WT thyroid tissue, while no differences were observed in *Mct10*^−/−^, *Ctsk*^−/−^/*Mct10*^−/−^, and *Ctsk*^−/−^/*Mct8*^−/y^ mice (Figure 6D). 

The analysis of thyrocyte areas that can, in principle, provide insights into cellular hypo- or hypertrophy revealed a significant increase in the average thyrocyte area in *Mct8*^−/y^, *Mct8*^−/y^/*Mct10*^−/−^, and *Ctsk*^−/−^/*Mct8*^−/y^/*Mct10*^−/−^ when compared to WT mice (Figure 6E). In contrast, *Mct10*^−/−^ mice showed a reduction in average thyrocyte area (Figure 6E, left panel). No change in average thyrocyte area was observed in *Ctsk*^−/−^/*Mct8*^−/y^ and *Ctsk*^−/−^/*Mct10*^−/−^ thyrocytes (Figure 6E, right panel). 

These data suggest that TSH receptor localization does not impact follicle, lumen, and thyrocyte areas or follicle numbers. The results further point to the notion that Mct8, rather than Mct10, is important in maintaining normal thyroid architecture on these parameters.

### 2.7. Enumerating Thyrocytes in Cathepsin K and/or TH Transporter Deficiency Reveals Absence of Mct10 as a Thyrocyte Survival Factor

**Figure 7:**
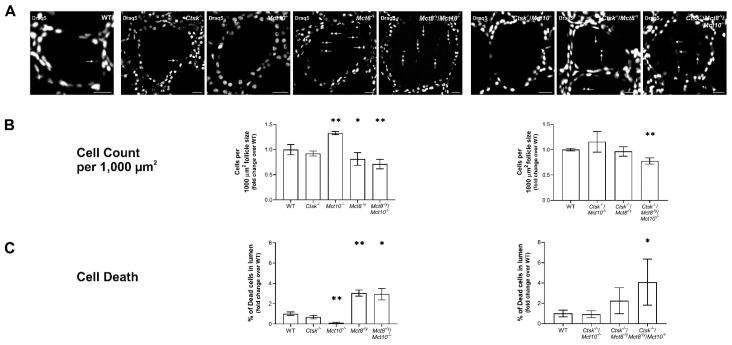
Thyrocyte counts and thyrocyte death rates in cathepsin K and/or TH transporter deficiency. Thyroid cryosections from indicated genotypes were incubated with Draq5^TM^ (white) to counter-stain cell nuclei (**A**). Dead cells shed into thyroid follicle lumina are indicated (**A**, arrows). Micrographs were analyzed by semi-automated morphometry using a Cell Profiler-based pipeline to determine thyrocyte counts. The number of cells per 1000 µm^2^ of follicle area and the percentage of intraluminal dead cells is displayed as bar graphs (**B** and **C**, respectively), both of which were significantly altered in the triple-deficient genotype (**B** and **C**, right panels). While *Mct10*^−/−^ mice showed increased cell counts when compared to WT controls, Mct8 single and Mct8/Mct10 double deficiency featured a contrasting decrease (**B**, left panel). Note that *Mct10*^−/−^ mice displayed significantly reduced cell death rates while lack of Mct8 function shows an opposite effect on thyrocyte survival (**C**, left panel). Animals analyzed: *n* = 3–6 per genotype. No. of follicles analyzed, in **B**: *n* = 1569, 703, 890, 1105, 614, 422, 552, and 630, and, in **C**: *n* = 1144, 830, 429, 511, 756, 445, 837, and 739, for WT, *Ctsk*^−/−^, *Mct10^−/−^, Mct8*^−/y^, *Mct8*^−/y^/*Mct10*^−/−^, *Ctsk*^−/−^/*Mct10*^−/−^, *Ctsk*^−/−^/*Mct8*^−/y^, and *Ctsk*^−/−^/*Mct8*^−/y^/*Mct10*^−/−^, respectively. Scale bars represent 20 µm. Note that the analyses were performed with different animal cohorts, therefore the data are depicted in comparison to the specific WT controls of each cohort and given as fold changes over WT controls as means ± SD. Levels of significance are indicated as * for *p* < 0.05, and ** for *p* < 0.01.

Since TSH receptor signaling, namely via the Gα_s_ pathway, regulates thyrocyte proliferation [25], we sought to determine Draq5^TM^-positive nuclei, i.e., thyrocyte numbers normalized to follicle area, in cryosections of WT, *Ctsk*^−/−^, *Mct10*^−/−^, *Mct8*^−/y^, *Mct8*^−/y^/*Mct10*^−/−^, *Ctsk*^−/−^/*Mct10*^−/−^, *Ctsk*^−/−^/*Mct8*^−/y^, and *Ctsk*^−/−^/*Mct8*^−/y^/*Mct10*^−/−^ mice (Figure 7). Cell profiler analysis revealed no changes in thyroids from *Ctsk*^−/−^, *Ctsk*^−/−^/*Mct10*^−/−^ or *Ctsk*^−/−^/*Mct8*^−/y^ mice in comparison to WT controls, whereas the *Mct8*^−/y^, *Mct8*^−/y^/*Mct10*^−/−^, and the triple-deficient genotypes showed a significant reduction in cell counts per 1000 µm^2^ follicle area (Figure 7A,B). In clear contrast, *Mct10*^−/−^ mice revealed significantly elevated cell numbers per 1000 µm^2^ follicle area (Figure 7A,B, left panels).

It is important to mention that the average cell counts per defined (1000 µm^2^) equatorial follicle section area is given by the sum of the number of proliferating and differentiated cells in the thyroid follicle epithelium, and the number of terminally differentiated thyrocytes that are shed into the follicle lumen where they remain as dead cell remnants [13,26]. Hence, it is plausible that altered numbers of thyrocytes per follicle area result from either changes in cell proliferation, cell death rates, or both. Therefore, Draq5^TM^-positive intraluminal cells remnants (Figure 7A, arrows) were counted and normalized to the total cell counts per thyroid mid-section. Results indicated the occurrence of dead cells in *Ctsk*^−/−^, *Ctsk*^−/−^/*Mct10*^−/−^, and *Ctsk*^−/−^/*Mct8*^−/y^ thyroid glands were comparable to WT controls, but elevated in *Mct8*^−/y^, *Mct8*^−/y^/*Mct10*^−/−^, and *Ctsk*^−/−^/*Mct8*^−/y^/*Mct10*^−/−^ showing three-fold and four-fold increases, respectively, in the proportion of dead cells (Figure 7C). Interestingly, the cell death rate was decreased in thyroid tissue of *Mct10*^−/−^ animals (Figure 7C, left panel) when compared to the WT controls. Together, we conclude that despite contrasting TSH receptor sub-cellular localization in the thyrocytes of *Mct8*^−/y^ (i.e., basolateral) vs. *Mct8*^−/y^/*Mct10*^−/−^ (i.e., vesicular), a similar result in terms of thyrocyte death rate was observed in these genotypes, implying that a functional link between TSH receptor localization and thyrocyte survival most likely does not exist, rather the self-toxicity due to lacking Mct8 function [6,8] prevails in these genotypes. Hence, the results indicated that Mct8 deficiency is associated with increased cell death, whereas a possible role of Mct10 in maintaining thyrocyte survival was observed.

## 3. Discussion

Studies have shown that despite the unusual pattern of TH serum concentrations in Mct8 deficiency [10,11,15,27], the HPT axis is deregulated differently in *Mct8*^−/y^ mice of different ages, namely TSH serum concentrations are significantly [10,27] or only moderately enhanced in *Mct8* deficiency [11,15,16]. We report in the present study that serum TSH concentrations in *Ctsk*^−/−^, *Ctsk*^−/−^/*Mct10*^−/−^, *Ctsk*^−/−^/*Mct8*^−/y^, and *Ctsk*^−/−^/*Mct8*^−/y^/*Mct10*^−/−^ mice showed no significant changes when compared to WT controls (see, Figure 1). Because we observed non-canonical TSH receptor localization in *Ctsk*^−/−^/*Mct8*^−/y^/*Mct10*^−/−^ mice (see, Figure 2), but neither in *Ctsk*^−/−^, *Ctsk*^−/−^/*Mct10*^−/−^ nor in *Ctsk*^−/−^/*Mct8*^−/y^ mice, we conclude that TSH receptor signaling within thyroid follicles is modulated by additional factors aside from serum TSH concentrations.

Hence, an important observation of the present study was an exclusive vesicular localization of the TSH receptor in *Mct10*^−/−^ and *Mct8*^−/y^/*Mct10*^−/−^ thyroid tissue, whereas *Ctsk*^−/−^/*Mct8*^−/y^/*Mct10*^−/−^ mice featured canonical basolateral TSH receptors in addition to their non-canonical vesicular localization. This led us to believe that signaling pathways involved in thyroid gland regulation are possibly altered in genotypes lacking TH transporters Mct8 and/or Mct10 particularly on a cathepsin K-deficient background, thereby resulting in altered thyroid function by TSH-independent auto-regulatory pathways. The latter is conceived because serum TSH concentrations were not altered in *Ctsk*^−/−^ alone or combined genotypes. Since thyroid dysfunctional states are accompanied by changes in tissue structure, we have examined whether a direct correlation between altered intrathyroidal TSH receptor signaling and altered thyroid morphology exists. Our data shows that Mct10 is important in maintaining TSH receptors in their canonical localization; however, non-canonical TSH receptor localization, possibly resulting in altered TSH receptor signaling, does not essentially lead to gross differences in thyroid gland architecture. A summary of the results achieved in this study and in complementation to our previous investigations is provided in Table 1.

### 3.1. Heteromerization of TSH Receptor and Mct8 Regulates Thyroid Gland Activity and Disruption in This Interaction Leads to Altered Thyroid Functional States

TSH receptor signals are mediated by coupling to Gα_s_ and Gα_q_ proteins [5,21,28]. Recently, the possible role of MCT8 in maintaining the balance between the Gα_s_ and Gα_q_ signaling pathways of TSH receptor signaling was described [22]. In vitro co-expression experiments in human cell culture models demonstrated molecular interactions between the TSH receptor and MCT8, which resulted in Gα_q_ signaling suppression [22]. Such a result of TSH receptor interaction with Mct8 in situ can be conceived, because we have observed the co-localization of both TSH receptor and Mct8 at the basolateral plasma membrane of mouse thyroid epithelial cells [3,7]. In addition, it has been suggested that the short-term TSH action requires the binding of two TSH molecules to TSH receptor homomers [29], which, in turn, may suggest that the interaction of TSH receptor with Mct8 reveals a new non-canonical mechanism of murine thyroid gland regulation, in that competition between formation of TSH receptor homomers and TSH receptor-Mct8 heterooligomers possibly regulates intrathyroidal TSH receptor signaling [3,22]. Such a regulatory mechanism would mean that the absence of Mct8 eliminates the competition for binding to TSH receptors, thus keeping TSH receptor homomers intact, thereby favoring short-term TSH action, i.e., Tg proteolysis by cathepsins, and thus activation of thyroid functional states. 

On the structural level, Gα_q_-induced enhancement of follicle activity is translated as increased epithelial heights, in that active thyrocytes feature a more prismatic shape rather than resting thyrocytes of a flat epithelium [7,13]. In the present study, we report that *Mct8*^−/y^/*Mct10*^−/−^ and *Ctsk*^−/−^/*Mct8*^−/y^/*Mct10*^−/−^ mice show increased epithelial extension, indeed indicative of enhanced thyroid activity in the absence of Mct8 and Mct10. 

Regarding the subcellular distribution of thyroidal cathepsins B, D, and L, analyzed as a proxy of Gα_q_ signaling, showed that while the total amounts of both extra- and intracellular cathepsins were enhanced in *Ctsk*^−/−^/*Mct8*^−/y^ and *Ctsk*^−/−^/*Mct8*^−/y^/*Mct10*^−/−^ mice, the proportional distribution of intraluminal (extra- and pericellular) over intracellular cathepsins remained unchanged. While this could mean that Gα_q_-mediated retrograde trafficking of cathepsins is unaltered in Mct8-deficient genotypes, another possible explanation would be that increased cathepsin secretion is balanced by enhanced cathepsin biosynthesis in *Ctsk*^−/−^/*Mct8*^−/y^ and *Ctsk*^−/−^/*Mct8*^−/y^/*Mct10*^−/−^ mice. Indeed, autophagy-induced lysosomal biogenesis is observed in these genotypes [6]. Therefore, future studies evaluating retrograde cathepsin trafficking in Mct8 deficiency upon inhibition of cathepsin biosynthesis, e.g., by RNA-interference approaches, would be important to distinguish Gα_q_ signaling from other effects like autophagy-mediated cathepsin upregulation. 

### 3.2. TSH Receptor Signaling Is Not Only Limited to the Cell Surface but also Persists within Intracellular Compartments

Classical TSH receptor localization, i.e., at the basolateral plasma membrane of thyrocytes, was either absent or only partially present in murine models lacking Mct10, which featured vesicular TSH receptor localization instead, such as observed in *Mct10*^−/−^, *Mct8*^−/y^/*Mct10*^−/−^, and *Ctsk*^−/−^/*Mct8*^−/y^/*Mct10*^−/−^ thyrocytes, indicating a role for Mct10 in keeping TSH receptors at their canonical position. Since sorting of TSH receptors to endosomes can mean that they are en route for either proteolytic degradation in lysosomes or for stimulating a new wave of cAMP production, we performed TSH receptor immunoblotting to evaluate receptor downregulation. We conclude that vesicular TSH receptors, at least those seen in *Ctsk*^−/−^/*Mct8*^−/y^/*Mct10*^−/−^ thyrocytes, remained intact since immunoblotting of triple-deficient mice thyroid lysates with TSH receptor-specific antibodies did not show prominent degradation fragments when compared to WT controls, suggesting persistent intracellular TSH receptor signaling in these genotypes. 

It is important to mention that endosomal TSH receptor signaling differs from that at the plasma membrane, both in terms of quality and quantity of downstream effects [17]. TSH-stimulated cAMP signaling from within endosomes continues to occur despite the removal of TSH, while signaling at the plasma membrane is reversibly halted when ligands dissociate. Gα_s_-mediated PKA activation induces vasodilator-stimulated phosphoprotein (VASP) phosphorylation resulting in actin depolymerization, which is implicated in the reuptake of Tg by thyrocytes for TH liberation within endo-lysosomes. However, inhibiting TSH receptor internalization leads to reduced VASP phosphorylation, thereby affecting actin cytoskeleton dynamics, indicating that intracellular cAMP signaling is important for Tg internalization and subsequent endocytic Tg degradation [17]. This points toward the notion that although serum TSH is unaffected in the triple-deficient murine model (this study), enhanced endo-lysosomal Tg proteolysis, as previously observed in this genotype [6], can be attributed to altered intrathyroidal TSH receptor signaling, i.e., increased Tg endocytosis and on-going signaling by vesicular TSH receptors. In support of this hypothesis, we have observed that mRNA levels of sortilin, a receptor that is expressed in a TSH-dependent manner and involved in Tg re-internalization [30], is significantly upregulated in *Ctsk*^−/−^/*Mct8*^−/y^/*Mct10*^−/−^ thyroid glands [6].

### 3.3. Altered TSH Receptor Signaling Does Not Necessarily Lead to Altered Thyroid Gland Morphology, but Absence of Mct10 Affects Thyrocyte Turnover

Keeping with the notion that endosomal TSH receptors couple to Gα_s_ and continue to mediate cAMP signaling, in the current study, we have evaluated whether the presence of TSH receptors in vesicles is associated with altered thyroid gland architecture. We report that thyroid follicle areas, luminal areas, and thyrocyte areas are not consistently affected in mice showing non-canonical vesicular TSH receptor localization, but rather these morphological parameters depend on Mct8 functionality. This is in accordance with our previous findings whereby we have shown that mice lacking trace-amine associated receptor 1 (Taar1), a GPCR that co-regulates thyroid function potentially by binding to TH metabolites in the thyroid follicle lumen, also feature vesicular TSH receptors but do not exhibit altered thyroid structural phenotypes [7]. Furthermore, this study reports Mct8 and Mct10 reveal opposing roles in maintaining thyrocyte numbers; while cell death rates are significantly reduced in *Mct10*^−/−^ mice, the *Mct8*^−/y^, *Mct8*^−/y^/*Mct10*^−/−^, *Ctsk*^−/−^/*Mct8*^−/y^, and *Ctsk*^−/−^/*Mct8*^−/y^/*Mct10*^−/−^ mice showed increased numbers of dead cells within the thyroid follicle lumen possibly due to thyrotoxic states seen in Mct8 deficiency [6,11]. In summary, aside from our previous reports that reveal the importance of cathepsin L for thyrocyte survival [13], in this study, we have established absence of Mct10 as an additional survival factor of thyrocytes. These data have set the stage to further investigate what specific molecular pathways of thyrocyte turnover are induced or repressed in Mct10 deficiency. 

Although the exact pathways of thyrocyte death remain unclear, studies have revealed that intrathyroidal lymphocytes play an important role in thyrocyte turnover [31]. One of the mechanisms of thyrocyte apoptosis involves autocrine or paracrine death receptor–ligand interactions, such as Fas/Fas-L, which results in the activation of caspases [32,33]. In addition, the apoptotic pathway is regulated by genes of the Bcl-2 family, some of which promote while others inhibit apoptosis by affecting mitochondrial integrity and regulating caspase activation [32,33,34,35]. In general, apoptosis of thyrocytes from normal and healthy thyroid tissue is a rare event. However, in certain autoimmune thyroid disorders such as Hashimoto’s thyroiditis and Graves’ disease or in thyroid cancers, the balance between thyrocyte cell death and survival is altered through the altered expression of apoptotic and anti-apoptotic genes [33,34,35]. For example, while altered Fas expression and downregulation of anti-apoptotic Bcl-2 genes has been implicated in increased thyrocyte death seen in Hashimoto’s thyroiditis, it has been suggested that TSH-mimicking TSH receptor antibodies in Graves’ disease inhibit Fas expression, thereby promoting anti-apoptotic mechanisms resulting in goiter formation and inducing death of intrathyroidal lymphocytes instead [32,36]. This suggests that interactions between different cell types within the thyroid are important in maintaining thyroid homeostasis [31]. 

In line with this notion, future studies will involve understanding in situ expression of pro- and anti-apoptotic genes in thyrocytes and to inspect mitochondrial functionality in TH transporter deficiencies. In addition, by using thyroid epithelial cell lines with transient inhibition of Mct10 function, such principal studies on cell cycle progression, apoptosis, and mitochondrial integrity can be carried out.

## 4. Materials and Methods

### 4.1. Murine Models

The study was performed using 5–8-month-old male *Ctsk*^−/−^, *Mct10*^−/−^, *Mct8*^−/y^, *Mct8*^−/y^/*Mct10*^−/−^, *Ctsk*^−/−^/*Mct10*^−/−^, *Ctsk*^−/−^/*Mct8*^−/y^, *Ctsk*^−/−^/*Mct8*^−/y^/*Mct10*^−/−^, and WT C57BL/6J mice. The generation and genotyping procedures have been described previously [6]. Mice were back-crossed to a congenic C57BL/6J background and housed in the transgenic animal facility of Jacobs University Bremen, Germany, licensed with the Authorities of the City State of Bremen (Senatorin für Gesundheit, Frauen und Verbraucherschutz der Hansestadt Bremen, Bremen, Germany) under registration numbers Az. 513-30-00/2-15-32 and Az. 0515_2040_15. Standardized conditions of light and dark cycles (12 h/12 h) with water and food ad libitum were maintained.

### 4.2. Serum Preparation, Dissection of Thyroid Tissue and Cryosectioning

Following euthanasia by CO_2_ inhalation, mice were perfused and the thyroid glands were excised as previously described [6]. For serum extraction, blood was taken from the right ventricles of the heart of 5–8-month-old male mice and allowed to clot by placing on ice for 30 min. Clotted material was removed and sera were cleared by centrifugation at 10,000× *g* for 10 min. Serum samples were stored at −20 °C until further use. For immunohistochemistry studies, thyroid tissue was fixed overnight at 4 °C in 4% paraformaldehyde in 200 mM HEPES, pH 7.4. Next, thyroid glands were incubated in Tissue Freezing Medium (Jung, through Leica Microsystems, Nussloch, Germany) overnight at 4 °C for cryopreservation and, subsequently, frozen in the gas phase of liquid nitrogen. Then, 5 µm thick transverse sections made using a cryostat (Leica CM1900, Leica Microsystems, Wetzlar, Germany) were thaw-mounted onto microscopic slides and stored at −20 °C. For protein biochemistry analyses, thyroid lobes were snap-frozen in liquid nitrogen and stored at −80 °C until subsequent use in tissue lysate preparation.

### 4.3. Indirect Immunofluorescence

Immunohistochemistry studies were performed as previously described [6]. Briefly, thyroid tissue sections washed overnight at 4 °C in calcium- and magnesium-free PBS (CMF-PBS), consisting of 0.15 M NaCl, 2.7 mM KCl, 1.5 mM NaH_2_PO_4_, and 8.1 mM Na_2_HPO_4_ at pH 7.4, were blocked with 3% bovine serum albumin (BSA; Carl Roth GmbH, Karlsruhe, Germany) in CMF-PBS for 1 h at 37 °C, and stained with specific primary antibodies diluted in 0.1% BSA in CMF-PBS overnight at 4 °C. The primary antibodies used in the current study were goat anti-mouse cathepsin B (1:100; #GT15047, Neuromics, through Acris, Herford, Germany), rabbit anti-human cathepsin D (1:10; #IM16, Calbiochem, through Merck Millipore, Darmstadt, Germany), goat anti-mouse cathepsin L (1:100; #GT15049, Neuromics), rabbit anti-mouse Laminin 1 + 2 (1:50; #ab7463, Abcam, Cambridge, UK), rabbit anti-mouse collagen IV (1:40; #CO20451, Novotech through BioLogo, Kronshagen, Germany), and mouse anti-human TSH receptor (1:100; #ab6047, Abcam). Secondary antibodies and counter-stains were combined and diluted in 0.1% BSA in CMF-PBS and incubated for 1 h at 37 °C. Secondary antibodies used in this study were Alexa Fluor 488-conjugated goat anti-rabbit IgG, rabbit anti-goat IgG, and goat anti-mouse IgG (1:200; Molecular Probes, Karlsruhe, Germany). Draq5^TM^ (10 µM; #DR05500, BioStatus Limited, Shepshed, Leicestershire, UK) served as a counter-stain to visualize nuclear DNA. To stain the cytoplasm of thyroid epithelial cells, tissue sections were incubated with HCS CellMask^TM^ Orange for 1 h at 37 °C (1:1000; #H32713, Molecular Probes). Microscopic slides were then washed in CMF-PBS and deionized water, mounted with an embedding medium composed of 33% glycerol and 14% Mowiol in 200 mM Tris-HCl, pH 8.5 (Hoechst AG, Frankfurt, Germany), and stored at 4 °C until further analysis by confocal laser scanning microscopy.

### 4.4. Image Acquisition

Immuno-stained thyroid cryo-sections were imaged using a Zeiss LSM 510 META laser scanning microscope equipped with argon and helium-neon lasers (Carl Zeiss Microscopy GmbH, Oberkochen, Germany). Confocal micrographs were obtained with a pinhole opening of 1 Airy unit and at a resolution of 1024 × 1024 pixels and stored in TIFF format. 8–10 images were arbitrarily chosen for each biological replicate and analyzed using LSM 5 software (version 3.2; Carl Zeiss Microscopy GmbH, Oberkochen, Germany). To study the thyroid gland morphometry and depict the follicle lumen sizes as a color-coded heat map, entire mid-sections of each of the thyroid lobes per genotype were imaged. The numbers of biological replicates per genotype and the numbers of thyroid follicles inspected in the study are indicated in the respective figure legends.

### 4.5. Automated Image Analysis

Image analyses were done using the open-source automated image analysis software Cell Profiler (version 3.1.9) [37], available from the Broad Institute at www.cellprofiler.org. Different pipelines P1, P2, and P3 consisting of a combination of different image-analysis modules were developed depending on the purpose and the intended read-outs of image analysis. The arrangement of each module and the details describing its function in the pipeline are given below.

#### 4.5.1. Fluorescence Intensity Measurements of Immunostained Thyroid Cryosections (Pipeline P1)

Cathepsin signal intensity exclusively in the extracellular lumen or over the epithelium was determined using P1 and as described previously [6]. Briefly, the module ‘ColorToGray’ was used to split the input RGB image into respective channels: Green (cathepsins B, D, or L), Red (CMO), and Blue (Draq5), and convert each into a gray scale output image, namely OrigGreen, OrigRed, and OrigBlue, respectively. The module ‘ApplyThreshold’ was applied to binarize the OrigRed image by applying a manual threshold value and create an output black and white image named as ‘ThresholdOrigRed’. The manual threshold value was set on the basis of the CMO staining, such that the signal is restricted to the epithelium, and does not bleed into the thyroid follicle lumen. The module ‘IdentifyPrimaryObjects’ was applied and using the OrigBlue image nuclei were identified by considering a user-defined diameter range that was set depending on the magnification and resolution of the input image. Next, total cathepsin (B, D, or L) fluorescence in the thyroid follicles was quantified by applying the module ‘MeasureImageIntensity’ for measuring the intensity of the OrigGreen image. To specifically quantify the intracellular signal, excluding the follicle lumen, the module ‘MaskImage’ was applied by using OrigGreen as the input image and ‘ThresholdOrigRed’ as the masking image. This resulted in an output image ‘IntraCellularCath’ whose intensity was determined using the module ‘MeasureImageIntensity’, thus enabling the fluorescence signals exclusive to the epithelia from all the thyroid follicles to be quantified. By subtracting the intracellular signal from the total signal measurements, the fluorescence intensity within the thyroid follicle lumen was determined. The signal measurements were normalized to the numbers of cells that were determined from Draq5™ staining for comparative analyses between different genotypes.

#### 4.5.2. Morphological Analysis of the Thyroid Gland (Pipelines P2 and P3)

**Figure 8:**
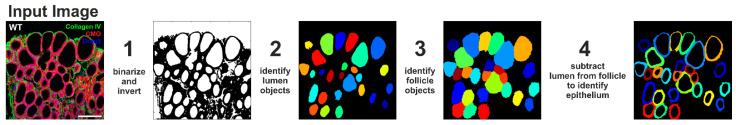
Morphological image analysis of mouse thyroid tissue using automated thyroid phenotyping tool. Thyroid cryo-sections were fixed with PFA and stained Collagen IV-specific antibodies (green), CMO (red), and Draq5^TM^ (blue) to visualize the nuclei, cytoplasm, and the extra-cellular matrix, respectively. A representative WT thyroid tissue section is shown (A). Determination of different architectural parameters was done using Cell Profiler-based pipeline P2. Merged greyscale images of all channels were binarized and inverted (**1**) to identify the follicle lumen objects (**2**). These objects were used as seeds, which were then propagated towards the Collagen IV-labelled follicle borders, to identify the follicle objects (**3**). Subtracting of the lumen area from the respective follicle area enabled identification of the epithelium (**4**). Areas of objects identified in steps 2 and 3 were used to determine the lumen and follicle areas, and epithelial extension. Nuclei identified by Draq5^TM^-staining were assigned to their respective follicle to determine cell counts per thyroid follicle. Area occupied by each epithelium object in step 4 was divided by the respective numbers of cells in it to determine the thyrocyte area.

To determine the follicle lumen area, epithelial height, thyrocyte area, follicle area, and numbers of cells per follicle, micrographs of thyroid cryo-sections stained with anti-Laminin 1+2 or Collagen IV antibody (green), CMO (red), and Draq5 (blue) were analyzed using the pipeline P2 (Figure 8) and as described previously [7]. The module ‘ColorToGrey’ was applied twice allowing for the splitting of the original three-channel input RGB image into separate greyscale images, as described for P1, and additionally the merging of these three channels to one single greyscale image ‘OrigGray’. Two modules of ‘ApplyThreshold’ were then used to apply a manual threshold to ‘OrigGreen’ and ‘OrigGray’ images and convert them to binary black and white output images, ‘ThresholdOrigGreen’ and ‘AllTissue’, respectively. Lower and upper limits of thresholding were adjusted according to the resolution of the input RGB image. It should be noted, however, that the same threshold was used for all images from the same batch of image acquisition. The module ‘Morph’ was applied two times on ‘ThreshOrigGreen’ to structure the green channel by closing gaps on a pixel per pixel basis and for skeletonizing the image, which produced the output image ‘ThreshOrigGreen_bin’. Next, the module ‘ImageMath’ was applied to invert the binary ‘AllTissue’ image, and the output image hence obtained was used to identify the lumen objects using the module ‘IdentifyPrimaryObjects’. In this module, the typical diameter of the lumen objects was set to 25–800 pixels, which must be adjusted depending on the magnification of the input RGB image. The sizes of the lumen objects thus identified by the module were measured using ‘MeasureObjectSizeShape’. In particular, the measurement ‘FormFactor’ as a measure of roundness for individual identified objects was determined. Next, the module ‘FilterObjects’ was used to exclude non-follicle objects based on the degree of roundness of an object. This filtering step was performed by manually setting a threshold value of 0.3, where 1.0 is a perfect circle. The filter can be decreased in case true follicles are removed from the selection. An optional module ‘EditObjectsManually’ was introduced to manually evaluate the identified objects and remove or add objects that have been identified wrongly. Subsequently, using the module ‘ImageMath’, the ‘ThreshOrigGreen_bin’ was subtracted from ‘AllTissue’ and the boundaries of the thyroid follicles were defined. The module ‘IdentifySecondaryObjects’ was applied to identify thyroid follicles by propagating from the lumen object to the follicle boundary. The number of pixels by which the primary objects (i.e., lumen) were to be expanded was manually set to 30 pixels to reach the follicle border. Once again, a manual editing step ‘EditObjectsManually’ was included to check for the appropriateness of the identified secondary objects (i.e., thyroid follicle). Next, ‘IdentifyTertiaryObjects’ was applied by subtracting the lumen objects from the follicle objects resulting in the epithelium objects. The module ‘RelateObjects’ makes child–parent relationship between objects and was used to assign each epithelium to its respective follicle. The module ‘CalculateMath’ was used to determine the lumen area, follicle area, and calculate the epithelial extension according to the mathematical equation described previously [7,13]. Nuclei were identified and counted using the ‘OrigBlue’ image as described above in P1. Subsequently, nuclei were assigned to the respective follicle by applying the ‘RelateObjects’ module. This was used to count the number of cells (thyrocytes) per follicle. By subtracting the lumen area from the follicle area, the area of the epithelium was obtained, which was divided by the number of cells per follicle to determine the thyrocyte area. 

To visually comprehend the heterogeneity in lumen areas, a color-coded heat map was generated using pipeline P3 and as described previously [7,14]. Micrographs were assembled by Paint.NET (created by Rick Brewster, 2004; registered trademark of dotPDN LLC; software available at http://www.getpaint.net/download.html) and used for analyzing the whole thyroid tissue mid-sections. In order to compare the heat maps from different genotypes, a standardized color-coding range with an upper-limit of lumen area was created by manually inserting a standard follicle with a cut-off area into each individual image to be analyzed. Thyroid follicle lumen objects and nuclei were identified as described above in P2. To identify the dead cell remnants in the follicle lumen, the module ‘MaskObjects’ was used to mask the identified nuclei with the identified lumen objects. Then, ‘RelateObjects’ was used to assign the identified dead cells to their respective parent follicle lumen and enumerate number of dead cells per follicle.

### 4.6. Preparation of Thyroid Tissue Lysate

Whole thyroid tissue lysates were prepared as described previously [6]. Briefly, deep-frozen thyroid tissue homogenized in PBS, pH 7.4, containing 0.5% Triton X-100 and protease inhibitors (0.02 M EDTA, 10 µM E64, 1 µM Pepstatin A, and 2 ng/mL Aprotinin) were incubated on ice for 1 h. Lysates were cleared by centrifugation at 16,000× *g* for 10 min at 4 °C, and stored at −20 °C. Quantification of protein concentrations in whole tissue lysates was performed by the Neuhoff assay as described [38]. Samples for SDS-PAGE were prepared by normalizing tissue lysates to equal protein amounts, i.e., 0.6 µg/µL protein per sample in sample buffer composed of 50 mM Tris-HCl (pH 7.6), 2.5% sodium dodecyl sulfate (SDS), 125 mM dithiothreitol, and 4 μM bromophenol blue), and heating for 5 min at 95 °C.

### 4.7. SDS-PAGE and Immunoblotting

Separation of thyroid tissue lysates on SDS-gels and immunoblotting was performed as detailed previously [6]. Briefly, protein samples were separated at 300 V and 50 mA on commercially available horizontal SDS Gradient 8–18 ExcelGel gels (GE Healthcare, Uppsala, Sweden) alongside the PageRuler^TM^ pre-stained protein ladder (#26616, Thermo Fisher Scientific, Schwerte, Germany). Next, proteins were transferred onto nitrocellulose membranes in a Novoblot Western Blotting chamber for 1 h at 30 V and 216 mA by semi-dry blotting method (GE Healthcare). Total protein per lane was determined by incubating the membranes with Ponceau S solution for 10 min at room temperature (#A2395, AppliChem, Darmstadt, Germany). Following blocking in 5% blotting-grade milk powder in PBS containing 0.3% Tween-20 (PBS-T) for 1 h at room temperature, membranes were incubated in primary antibodies, diluted in PBS-T, overnight at 4 °C. Primary antibodies used for immunoblotting was mouse anti-human TSH receptor (1:500; #ab27974, Abcam). After washing the membranes with PBS-T for 30 min to remove unbound primary antibodies, membranes were incubated in horseradish peroxidase (HRP)-conjugated goat anti-mouse IgG (H + L) secondary antibodies (Southern Biotech, Birmingham, USA) that were diluted in PBS-T containing 2.5% milk powder at 1:5000 for 1 h at 25 °C. Then, membranes were washed in PBS-T for 30 min and incubated with ECL Western blotting substrate for 3 min. Membranes were then exposed onto CL-XPosure^TM^ films (Pierce via Thermo Fisher Scientific) to visualize the immunolabeled proteins by enhanced chemiluminescence. Exposed films were scanned using a transmitted-light scanner (Desk Scan II version 2.9, Hewlett-Packard Co., Palo Alto, California, CL, USA). Band densitometry analysis was performed using Image Studio Lite version 5.2 (LI-COR Biosciences GmbH, Bad Homburg, Germany).

### 4.8. Measurement of Serum TSH Concentrations

Blood serum samples of individual mice were used for evaluation of TSH in duplicates or triplicates by use of a mouse TSH-specific competitive inhibition enzyme immunoassay (#CEA463MU, Cloud-Clone Corp. via Antibodies Online, Aachen, Germany) according to the instructions provided by the manufacturer and as described previously [7,39,40,41]. Briefly, 50 µL of standards with known TSH concentration or serum samples were incubated with biotin-labeled TSH in a microplate that was pre-coated with an antibody specific to mouse TSH to allow for competitive inhibition reaction to occur. Then, unbound conjugate was washed off and incubated with HRP-conjugated avidin. Next, the substrate solution provided by the manufacturer was added and absorbance was measured at 450 nm using a Tecan Infinite M1000 Pro instrument (Grödig, Salzburg, Austria). The number of biological replicas were *n* = 6, 4, 5, 3, 8 for WT, *Ctsk*^−/−^, *Ctsk*^−/−^/*Mct10*^−/−^, *Ctsk*^−/−^/*Mct8*^−/y^, and *Ctsk*^−/−^/*Mct8*^−/y^/*Mct10*^−/−^, respectively. The mouse TSH-specific ELISA kit used in this study had the following coefficients of variation (CV), namely, CV < 10% for intra-assay and CV < 12% for inter-assay values. The detection range of the mouse TSH ELISA kit is 6.4–4000 pg/mL with a sensitivity minimum of 2.2 pg/mL. 

### 4.9. Statistical Analysis

Band densitometry from immunoblotting and quantification of fluorescence signals from immunohistochemistry are presented as means ± standard deviations. All measurements are displayed as fold changes over wild-type controls to depict genotypic effects. Levels of significance were determined by performing one-way ANOVA with Dunnett’s correction for multiple comparisons using GraphPad Prism^TM^ (version 8.02; GraphPad Software Inc., San Diego, CA, USA), *p* values below 0.05 were considered statistically significant. 

## Figures and Tables

**Table 1:** Summary of phenotypic changes determined in cathepsin K and/or thyroid hormone transporter deficiency. Assessed parameters are listed with indications to serving as proxy for Gα_q_ or Gα_s_, respectively. TSH receptor distribution is basolateral for canonical and vesicular for non-canonical localization, representative of Gα_q_ short-term or Gα_s_ long-term signaling, and prolonged Gα_s_ signaling, respectively. Changes are depicted for the indicated genotype vs. wild type as = for no change, ↑ for increase, and ↓ for decrease.

Parameter	Proxy for Gα_q_ or Gα_s_	*Ctsk* ^−/−^	*Mct10* ^−/−^	*Mct8* ^−/y^	*Mct8*^−/y^/ *Mct10*^−/−^	*Ctsk*^−/−^/ *Mct10*^−/−^	*Ctsk*^−/−^/ *Mct8*^−/y^	*Ctsk*^−/−^/ *Mct8*^−/y^/ *Mct10*^−/−^
Serum TSH		=	= [11]	= ↑ [10,11,27]	↑ [11]	=	=	=
TSH receptor localization		basolateral	vesicular	basolateral	vesicular	basolateral	basolateral	basolateral and vesicular
EExt	Gα_q_	= [14]	=	=	↑	=	=	↑
extra- vs. intracellular cathepsins	Gα_q_	↑ [13]	= [8]	= [8]	= [8]	=	=	=
Tg degradation	Gα_q_	= [6]	= [8]	↑ [8]	↑ [8]	= [6]	↑ [6]	↑ [6]
Tg protein	Gα_s_	= [6]				↓ [6]	= [6]	= [6]
Tg cross-linkage	Gα_s_	= [6]	= [8]	↓ [8]	↓ [8]	= [6]	= [6]	= [6]
Tg glycosylation	Gα_s_	= [6]				↓ [6]	↓ [6]	↓ [6]
cell. Cath B	Gα_s_	= [13]	↑ [8]	↑ [8]	↑ [8]	=	↑	↑
cell. Cath D	Gα_s_	= [13]	= [8]	= [8]	↑ [8]	=	↑	↑
cell. Cath L	Gα_s_	↑ [13]	↑ [8]	↑ [8]	↑ [8]	=	↑	↑
Follicle no.	Gα_s_	= [14]	=	=	=	=	↑	=
Follicle area	Gα_s_	= [14]	=	↑	↑	=	=	↑
Lumen area	Gα_s_	= [14]	=	↑	↑	=	=	↑
TC area	Gα_s_	=	↓	↑	↑	=	=	↑
Cell no. per 1000 µm^2^	Gα_s_	=	↑	↓	↓	=	=	↓
Cell Death	Gα_s_	=	↓	↑	↑	=	=	↑

Abbreviations used stand for cell. = cellular, no. = numbers, and TC = thyrocyte.

## Data Availability

Not applicable.

## Supplementary Materials

The following are available online at https://www.mdpi.com/article/10.3390/ijms22115776/s1.
