# Supplementary figures and images for: The Amino Acid Transporter Mct10/Tat1 Is Important to Maintain the TSH Receptor at Its Canonical Basolateral Localization and Assures Regular Turnover of Thyroid Follicle Cells in Male Mice
Source: Int J Mol Sci. 2021 May 28;22(11):5776. doi: 10.3390/ijms22115776 (PMC8198332; doi:10.3390/ijms22115776)

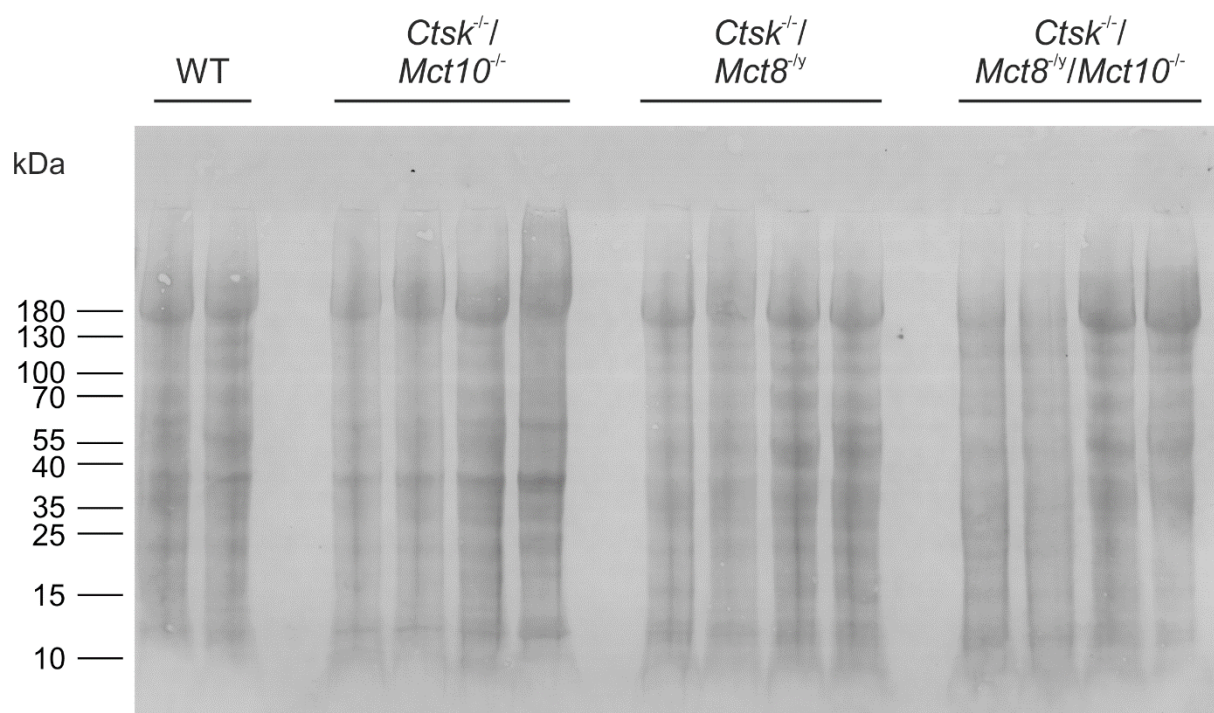

Supplementary Figure S1. Ponceau-stained loading control corresponding to Figure 3A.

Supplement: Supplementary file 1 [file ijms-22-05776-s001.zip › ijms-1224504-supplementary.pdf]
